# Evaluation and spatiotemporal evolution of veterinary talent competitiveness: a new perspective of veterinary education

**DOI:** 10.3389/fvets.2024.1415753

**Published:** 2024-11-29

**Authors:** Xianhang Xu, Mohd Anuar Arshad, Yugang Jian, Arshad Mahmood, Mengdie Dong

**Affiliations:** ^1^School of Management, Chongqing Institute of Engineering, Chongqing, China; ^2^School of Management, Universiti Sains Malaysia, Penang, Malaysia; ^3^School of Business and Economics, University of Wuppertal, Wuppertal, Germany

**Keywords:** veterinary, talent competitiveness, veterinary education, evaluation index, spatiotemporal evolution

## Abstract

**Introduction:**

Improving the quantity and quality of veterinary talent cultivation is an urgent issue to address in current veterinary education and is key to enhancing veterinary talent competitiveness. Starting from an industrial perspective, the introduction of scientific analytical methods for studying veterinary talent competitiveness offers a new view on veterinary education and helps to improve the quality of veterinary education and talent cultivation.

**Methods:**

This study develops a veterinary talent competitiveness evaluation index based on the characteristics of veterinary talent. It proposes a visual method to analyze the level and spatiotemporal evolution of veterinary talent competitiveness using the Entropy method and ArcGIS tools, with Western China as a case study. Data is collected from 12 regions in Western China, spanning 2015 to 2021.

**Results:**

The results show that the method not only evaluates the current state of veterinary talent competitiveness but also considers temporal and spatial evolution, achieving good evaluation effectiveness and high accuracy, thereby guiding the improvement of veterinary education and talent cultivation.

**Discussion:**

Based on the research findings, the study suggests improving the quality of veterinary education and talent cultivation through measures such as strengthening talent resource development, addressing regional imbalances, and promoting spatial integration to achieve a virtuous cycle between veterinary education and industrial development.

## 1 Introduction

Animal husbandry is vital to people's livelihood (1), serving as a strategic industry that ensures food security and the wellbeing of residents (2). Veterinarians are an indispensable part of animal husbandry (3), playing a crucial role in ensuring animal health, improving production efficiency, securing food safety, and supporting public health (4). China is the largest animal husbandry producer in the world (5). With the rapid development of animal husbandry, the demand for veterinary professionals has significantly increased (6). However, issues such as the insufficient number of veterinarians and the uneven quality of veterinary talent have begun to hinder the sustainable development of this industry. Talent is the primary resource (7), and the progress of any industry is deeply linked to the strategic distribution of skilled talent (8), which cannot be separated from veterinary talent cultivation. Improving the quantity and quality of veterinary talent cultivation has become an urgent issue that needs to be addressed in current veterinary education (9). Veterinary education forms the foundation for cultivating veterinary talent (10), and the quantity and quality of talent cultivation directly determine veterinary talent competitiveness. It requires veterinary educational institutions to provide high-quality education to cultivate talents that meet the demands of the modern industry, and it necessitates continuous evaluation and monitoring of veterinary talent competitiveness. Furthermore, with changing societal needs and advancements in veterinary science, veterinary education faces many new challenges and opportunities (11). It calls for a new perspective to review and adjust the strategies and content of veterinary education, to cultivate veterinary professionals who can adapt to future changes.

Veterinary talent competitiveness is essentially the research category of industrial talent competitiveness. This competitiveness is a complex concept, encompassing factors like the scale, structure, and efficiency of talent within an industry and the investment, policies, and environmental factors influencing talent development (12). This concept is manifested as the comprehensive competitiveness of a country or region's specific industry regarding the quantity, quality, structure, support, and environment of talent (13). Scholars have utilized various methods such as Analytic Hierarchy Process (AHP), Principal Component Analysis (PCA), Entropy, Neural Network, Gray analysis, and Structural Equation Modeling (SEM) to develop talent competitiveness evaluation indexes (14–17), which are used to assess the talent competitiveness across different countries, regions, and industries.

Regarding national and regional talent competitiveness research, the Global Talent Competitiveness Index (GTCI) released by the European Institute of Business Administration (INSEAD) has gained widespread recognition. This index analyzes the talent competitiveness of various countries primarily from talent input and output (18). Based on GTCI, many scholars have developed talent competitiveness evaluation indexes to study talent competitiveness in countries like Romania, Lithuania, India, the Republic of Moldova, China, Ukraine, and others (19–25). Based on their understanding, scholars have studied national and regional talent competitiveness from the dimensions of talent resources, talent input, talent efficiency, talent environment, etc. (15, 26–29).

In exploring industrial talent competitiveness, scholars have delved into various sectors. These include Nigeria's banking industry, the green chemical technology industry in Eastern China, Greece's shipping industry, Shenzhen's chip industry, Chongqing's intelligent industry, and Liaoning's manufacturing industry (30–34). As a particular type of industrial talent competitiveness, some scholars have also studied scientific and technological talent competitiveness (16, 35–37).

Currently, interdisciplinary research has become a trend in various fields. GIS technology can perform spatial analysis and visualization display (38, 39), scholars have increasingly conducted various competitiveness studies using GIS technology, mainly involving tourism competitiveness (40–42), logistics competitiveness (43, 44), cultural competitiveness (45), environmental competitiveness (46), trade competitiveness (47), regional competitiveness (48–51), industrial competitiveness (52–55), and corporate competitiveness (56, 57).

The literature shows that current research on talent competitiveness primarily focuses on national, regional, and scientific and technological talents, with limited results related to industries. Particularly, there is a significant gap in research concerning the veterinary talent competitiveness. Meanwhile, the research on the competitiveness of GIS technology has involved multiple aspects, but there is little research on talent competitiveness. Therefore, from the perspective of industry, constructing an evaluation index based on the characteristics of veterinary talent to measure the level of veterinary talent competitiveness, combining GIS technology to analyze its spatiotemporal evolution, and providing a new perspective for the formulation of veterinary education strategies, which has theoretical and practical implications. The powerful spatial data analysis of GIS technology provides a new perspective and method for the study. It provides effective support for understanding the spatial evolution of competitiveness and can promote the development of interdisciplinary research such as veterinary medicine, economics, and geography, which will be an interesting exploration.

According to the above analysis, the aims of this study are as follows: Firstly, to develop a veterinary talent competitiveness evaluation index based on the characteristics of veterinary talent; Secondly, to utilize the Entropy method and GIS technology to analyze the level and spatiotemporal evolution of veterinary talent competitiveness in Western China; Thirdly, to propose suggestions for veterinary education from the perspective of talent competitiveness.

## 2 Methods

### 2.1 Research area

**Figure 1:**
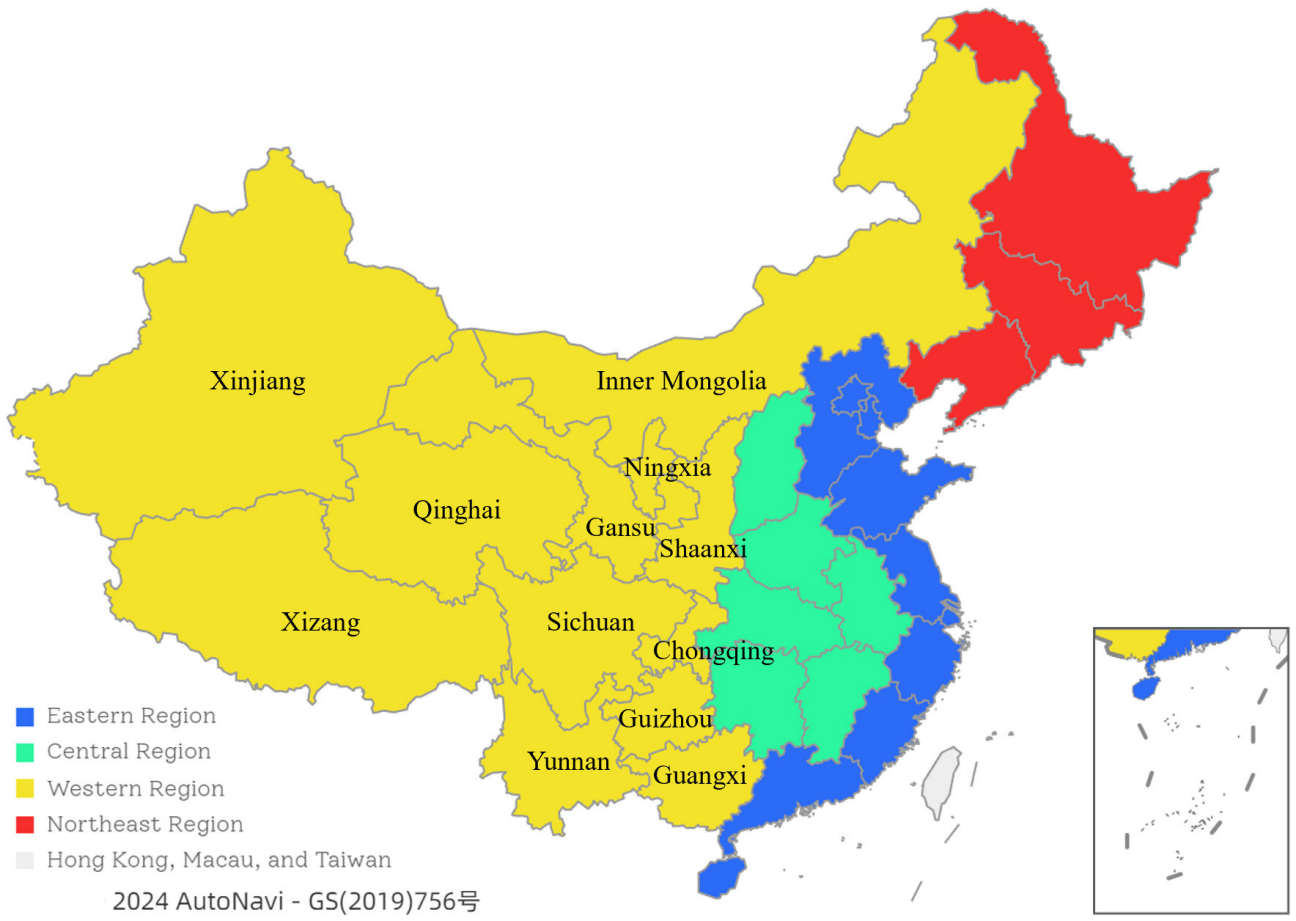
The regions of China.

The scope of this study covers Western China, including 12 provinces, municipalities, and autonomous regions (see Figure 1). Western China occupies 70.6% of China's land area and 84.2% of its grassland area. All four of China's major pastoral areas are in Western China, making it a focal point for developing animal husbandry. Western region has abundant animal husbandry resources and gathers most of the veterinary talent in China. Therefore, selecting Western China for research is representative and feasible.

### 2.2 Data sources

This study evaluates the veterinary talent competitiveness in 12 regions in Western China, spanning 2015 to 2021. The data are sourced from the “China Statistical Yearbook” (58), “China Animal Husbandry and Veterinary Yearbook” (59), “China Rural Statistical Yearbook” (60), “China Statistical Yearbook on Science and Technology” (61), and “National Education Fund Implementation Statistics Announcement” (62).

### 2.3 Evaluation method

This study develops a veterinary talent competitiveness evaluation index and applies the Entropy method for evaluation.

#### 2.3.1 Development of evaluation index

From the perspective of veterinary education and veterinary talent cultivation, this study draws on scholars' selection of dimensions and indicators for talent competitiveness evaluation. It explores a conceptual model from four dimensions: talent resources, talent investment, development support, and development environment.

The talent resources dimension reflects the number, structure, and contribution of talents in the industry within the region (63, 64), representing the effect of veterinary talent cultivation and the current level of talent competitiveness, which are the key to ensuring industry development (65, 66). Given the inconsistency in the statistical caliber of veterinary talent in different regions, this study uses data from local township animal husbandry and veterinary stations. The talent investment dimension reflects the comprehensive investment in talent development within a certain period in the region (67, 68), including the investment in veterinary education, representing the importance that governments and residents attach to activities such as education, technology, and innovation, which is directly related to potential talent cultivation (69, 70). The development support dimension reflects the external support forces of the region in attracting, selecting, developing, and retaining talents, representing the support of education, technology, culture, and health affairs, and are directly related to the sustainable development of talents (71–73), which will act on the process of veterinary education and veterinary talent cultivation. The development environment dimension reflects the economic development, industry development, and people's living standards of rural areas in the region (74–76), representing the impact of the development environment on the work and life of talents. It has become an important factor and potential attraction for attracting veterinary talent.

According to the conceptual model above, this study learns from scholars' selection of talent competitiveness evaluation indicators and develops an evaluation index (see Table 1).

**Table 1:** Evaluation index of veterinary talent competitiveness.

**Dimension**	**Indicator**	**Unit**	**Direction**	**References**
Talent resources	Number of personnel of township animal husbandry and veterinary station	People	+	(16, 27, 29, 37)
	Proportion of the number of senior technical titles of township animal husbandry and veterinary station	%	+	
	Proportion of personnel with master's degree or above in township animal husbandry and veterinary station	%	+	
	Per capita output value of township animal husbandry and veterinary station	10,000 CNY	+	
Talent investment	Proportion of education expenses in general public budget expenditures	%	+	(18, 20, 26–28)
	Intensity of local financial investment in science and technology	%	+	
	Per capita education, culture, and entertainment expenditure of rural residents	CNY	+	
	General public budget agricultural, forestry, and water input per hectare of agricultural land	CNY	+	
Development support	Number of students in higher education per 100,000 people	People	+	(16, 22, 31, 33, 77)
	Number of R&D employees per 10,000 people	People	+	
	Number of cultural service institutions per 100,000 people	Unit	+	
	Number of beds in medical and health institutions per 1,000 people	Bed	+	
Development environment	Total power for agricultural machinery per 10,000 rural people	KWH	+	(14, 15, 29, 30, 36, 37)
	Per capita electricity consumption of rural residents	KWH	+	
	Per capita disposable income of rural residents	CNY	+	
	Personnel salary of township animal husbandry and veterinary station	CNY	+	

CNY, Chinese Yuan; KWH, Kilowatt-hour.

#### 2.3.2 Talent competitiveness evaluation

The weighting of evaluation indicators is the key to building a talent competitiveness evaluation index. Considering the subjective arbitrariness of the subjective weight method and the significant deviation it brings (78), this study uses the Entropy method to weight the indicators and evaluate the veterinary talent competitiveness in different regions.

**(1) Data standardization processing**

Constructing the original data matrix of indicators:


$$X=\left[\begin{array}{ccc} X_{11} & \cdots & X_{1j}\\ \vdots & \ddots & \vdots \\ X_{i1} & \cdots & X_{ij} \end{array}\right]$$

Here, X_ij_ is the original value, representing the j-th indicator of the i-th region, and i = 1, 2, …, 12, j = 1, 2, …, 16.

Using the extreme value method to process positive and negative indicators separately, and Y_ij_ is the standardized value.

Positive indicator processing:


(1) $$\begin{array}{l} Y_{ij}=\frac{X_{ij}-\min\left(X_{ij}\right)}{\max\left(X_{ij}\right)-\min\left(X_{ij}\right)} \end{array}$$

Negative indicator processing:


(2) $$\begin{array}{l} Y_{ij}=\frac{max\left(X_{ij}\right)-X_{ij}}{\max\left(X_{ij}\right)-min\left(X_{ij}\right)} \end{array}$$

To avoid zero values, perform translation processing on the processed data:


(3) $$Y_{ij}^{\prime }=\frac{Y_{ij}+\alpha }{1+\alpha }$$

Here, to preserve the original data pattern, take α = 0.001.

After processing, the standardized data matrix is finally obtained:


$$Y=\left[\begin{array}{ccc} Y_{11}^{\prime } & \cdots & Y_{1j}^{\prime }\\ \vdots & \ddots & \vdots \\ Y_{i1}^{\prime } & \cdots & Y_{ij}^{\prime } \end{array}\right]$$

**(2) Calculate the proportion of each measurement indicator**


(4) $$P_{ij}=Y_{ij}^{\prime }/\sum_{i=1}^{n}Y_{ij}^{\prime }$$

**(3) Calculate the information entropy of each measurement index**


(5) $$\begin{array}{l} E_{j}=-\frac{1}{lnn}\overset{n}{\underset{i=1}{\sum }}P_{ij}\ln\;P_{ij} \end{array}$$

**(4) Calculate the weight of each evaluation indicator**


(6) $$\begin{array}{l} W_{j}=\frac{1-E_{j}}{m-\overset{m}{\underset{j=1}{\sum }}E_{j}} \end{array}$$

Among them, the range of W_j_ is [0, 1], j=1, 2, …, m.

**(5) Calculate the comprehensive veterinary talent competitiveness**


(7) $$V_{i}=10\times \sum_{i=1}^{m}W_{j}Y_{ij}^{\prime }$$

Among them, the V_i_ value reflects the strength of veterinary talent competitiveness in the region. The larger the V_i_ value, the higher the veterinary talent competitiveness in the region.

### 2.4 Spatial analysis method

This study analyzes the spatiotemporal evolution of veterinary talent competitiveness using ArcGIS software. Based on the principle of quartile difference in statistics, this study grades the total scores of veterinary talent competitiveness in Western China from 2015 to 2021 according to four levels. Then, it employs ArcGIS software to visualize the spatial pattern of competitiveness and intuitively display the spatial distribution of veterinary talent competitiveness.

Spatial autocorrelation analysis is an essential method in geographical research (79). By measuring the degree of aggregation of spatial unit attribute values, the spatial dependence between an object and its spatial position is explored, which is utilized to analyze the statistical distribution of spatial data (80). A positive correlation indicates that a spatial unit's attribute values change has the same trend as its neighboring units, while a negative correlation indicates the opposite (81). To better reveal the spatial changes in veterinary talent competitiveness, this study analyzes the spatial correlation of regions through Global Moran's I.


(8) $$\begin{array}{l} I=\frac{\overset{n}{\underset{i=1}{\sum }}\overset{n}{\underset{j=1}{\sum }}W_{ij}\left(X_{i}-\bar{X}\right)\left(X_{j}-\bar{X}\right)}{S^{2}\overset{n}{\underset{i=1}{\sum }}\overset{n}{\underset{j=1}{\sum }}W_{ij}} \end{array}$$

Here, I is the value of Moran's I, with a range of [−1,1]. A value exceeding 0 signifies a positive correlation, a value below 0 denotes a negative correlation, and a value exactly at 0 indicates no correlation. X_i_ and X_j_ are the values of variables i and j in regions, $\bar{X}$ is the mean value, n is the province number, s^2^ is the variable's variance, and W_ij_ is the spatial weight between regions i and j.

To ensure the accuracy of Moran's I, Z-Score is commonly utilized for significance testing (82). The calculation formula is:


(9) $$\begin{array}{l} Z\left(I\right)=\frac{\left[I-E\left(I\right)\right]}{\sqrt{VAR\left(I\right)}} \end{array}$$

Here, Z(I) represents the level of significance, E(I) is the expectation of the I value, and VAR(I) is the variance of the I value. A 95% confidence interval is used for significance testing. When |Z| > 1.96, it indicates a significant spatial autocorrelation. When |Z| < 1.96, it indicates a random distribution of observed values. When Z > 1.96, there is a positive correlation and a clustered distribution. When Z < −1.96, there is a negative correlation and a discrete distribution.

Global Moran's I is difficult to reflect the degree of correlation between a region and its surrounding provinces and cities. Consequently, the study integrates the Local Indicators of Spatial Association (LISA) analysis technique. This method is employed to break down the overall spatial correlation coefficient into individual spatial autocorrelations for each specific region, facilitating a more localized and detailed spatial analysis (83). Divide the LISA aggregation map into four types of regions, namely high value regions surrounded by adjacent high values (H-H), high value regions surrounded by adjacent low values (H-L), low value regions surrounded by adjacent high values (L-H), and low value regions surrounded by adjacent low values (L-L). H-H and L-L represent spatial aggregation, while H-L and L-H represent spatial dispersion or differentiation. The formula of LISA Moran's I is:


(10) $$\begin{array}{l} I_{L}=\frac{\left(X_{i}-\bar{X}\right)}{S^{2}}\overset{n}{\underset{j=1}{\sum }}W_{ij}\left(X_{j}-\bar{X}\right) \end{array}$$

## 3 Results

### 3.1 Evaluation index weighting

According to Equations 1–6 and the data of western region from 2015 to 2021, the evaluation dimensions and indicators selected in Section 2 were weighted (see Table 2).

**Table 2:** Weights of veterinary talent competitiveness evaluation dimensions and indicators.

**Dimension**	**Weight**	**Indicator**	**Entropy value**	**Weight**
Talent resources	0.3285	Number of personnel of township animal husbandry and veterinary station	0.9382	0.0669
		Proportion of the number of senior technical titles of township animal husbandry and veterinary station	0.8763	0.1338
		Proportion of personnel with master's degree or above in township animal husbandry and veterinary station	0.9356	0.0697
		Per capita output value of township animal husbandry and veterinary station	0.9462	0.0581
Talent investment	0.1838	Proportion of education expenses in general public budget expenditures	0.9586	0.0448
		Intensity of local financial investment in science and technology	0.9503	0.0537
		Per capita education, culture, and entertainment expenditure of rural residents	0.9804	0.0212
		General public budget agricultural, forestry, and water input per hectare of agricultural land	0.9408	0.0640
Development support	0.2379	Number of students in higher education per 100,000 people	0.9599	0.0434
		Number of R&D employees per 10,000 people	0.9376	0.0675
		Number of cultural service institutions per 100,000 people	0.9115	0.0957
		Number of beds in medical and health institutions per 1000 people	0.9710	0.0313
Development environment	0.2499	Total power for agricultural machinery per 10,000 rural people	0.9112	0.0961
		Per capita electricity consumption of rural residents	0.9545	0.0492
		Per capita disposable income of rural residents	0.9645	0.0384
		Personnel salary of township animal husbandry and veterinary station	0.9389	0.0661

From the four dimensions of veterinary talent competitiveness in Table 2, the weight of the talent resources dimension is 0.3285, and the average weight of a single indicator is 0.0821. The weight of the talent investment dimension is 0.1838, and the average weight of a single indicator is 0.0459. The weight of the development support dimension is 0.2379, and the average weight of a single indicator is 0.0595. The weight of the development environment dimension is 0.2499, and the average weight of a single indicator is 0.0625. From the weight of 16 indicators to evaluate the level of veterinary talent competitiveness, the proportion of the number of senior technical titles of township animal husbandry and veterinary station (0.1338), the total power for agricultural machinery per 10,000 rural people (0.0961), the number of cultural service institutions per 100,000 people (0.0957), the proportion of people with master's degree or above in township animal husbandry and veterinary station (0.0697), the number of R&D practitioners per 10,000 people (0.0675), the number of personnel of township animal husbandry and veterinary station (0.0669), the personnel salary of township animal husbandry and veterinary station (0.0661), and the general public budget agricultural, forestry, and water input per hectare of agricultural land are greater than the average (0.0625). The talent resources dimension has the highest weight, and three indicators are above average (0.0625), indicating that it has a significant impact on veterinary talent competitiveness. The weight of development support and development environment dimensions takes second place, indicating their impact on veterinary talent competitiveness is moderate. The weight of the talent investment dimension is the lowest, indicating its impact on veterinary talent competitiveness is relatively small.

### 3.2 Measurement of veterinary talent competitiveness

According to Equation 7, this study calculates the comprehensive index of veterinary talent competitiveness of 12 regions in Western China. The results are ranked to quantitatively reflect the veterinary talent competitiveness in each region from 2015 to 2021 (see Table 3).

**Table 3:** Evaluation of veterinary talent competitiveness (2015–2021).

**Region**	**2015**		**2016**		**2017**		**2018**		**2019**		**2020**		**2021**		**Average**	
	**Value**	**Rank**	**Value**	**Rank**	**Value**	**Rank**	**Value**	**Rank**	**Value**	**Rank**	**Value**	**Rank**	**Value**	**Rank**	**Value**	**Rank**
Chongqing	3.23	1	3.78	1	4.26	1	4.93	1	4.87	1	5.42	1	5.81	1	4.61	1
Sichuan	2.73	3	2.92	3	3.35	4	3.68	4	3.93	5	4.30	5	4.86	5	3.68	4
Guizhou	1.82	9	2.17	7	2.59	6	2.87	6	3.11	6	3.39	10	3.64	8	2.80	7
Yunnan	2.00	6	2.67	4	3.18	5	3.63	5	4.02	4	4.47	3	4.96	3	3.56	5
Xizang	1.04	12	0.62	12	1.19	12	1.47	12	1.83	12	1.93	12	2.68	12	1.54	12
Shaanxi	2.98	2	3.19	2	3.61	2	3.76	3	4.61	2	4.75	2	4.93	4	3.98	2
Gansu	1.91	8	1.97	9	2.21	9	2.45	10	2.80	8	3.70	6	3.49	9	2.65	9
Qinghai	1.13	11	1.29	11	2.03	11	2.04	11	2.41	11	3.48	8	3.15	11	2.22	11
Ningxia	2.47	4	2.53	5	3.52	3	3.92	2	4.14	3	4.40	4	5.03	2	3.72	3
Xinjiang	1.99	7	2.12	8	2.20	10	2.47	9	2.77	9	3.63	7	3.68	7	2.69	8
Inner Mongolia	2.25	5	2.34	6	2.55	7	2.81	7	3.02	7	3.40	9	3.94	6	2.90	6
Guangxi	1.75	10	1.88	10	2.23	8	2.48	8	2.70	10	3.05	11	3.46	10	2.51	10
Mean	2.11	—	2.29	—	2.74	—	3.04	—	3.35	—	3.83	—	4.14	—	3.07	—

The score of veterinary talent competitiveness in Western China has continued to rise from 2.11 in 2015 to 4.14 in 2021, showing an upward trend year by year. The score of talent competitiveness in the veterinary industry in various regions is relatively low, and the overall level needs to be improved, with uneven regional development levels. Chongqing has always had the strongest veterinary talent competitiveness. It has risen from 3.23 in 2015 to 5.81 in 2021, ranking first for 7 consecutive years. Shaanxi has continued to rise from 2.98 in 2015 to 4.93 in 2021, ranking second on average. Ningxia has risen from 2.47 in 2015 to 5.03 in 2021, ranking third on average. Sichuan has risen from 2.73 in 2015 to 4.86 in 2021, ranking fourth on average. Yunnan has risen from 2.00 in 2015 to 4.96 in 2021, ranking fifth on average. The average score of these five regions is greater than the average level in the western region, indicating relatively strong talent competitiveness. The average score of Inner Mongolia, Guizhou, Xinjiang, Gansu, Guangxi, Qinghai, Xizang, and other regions ranks sixth to twelfth, respectively. The average score of these seven regions is lower than the average level of the western region, and the talent competitiveness is relatively weak.

### 3.3 Spatiotemporal evolution of veterinary talent competitiveness

#### 3.3.1 Spatial differentiation and evolution of veterinary talent competitiveness

**Figure 2:**
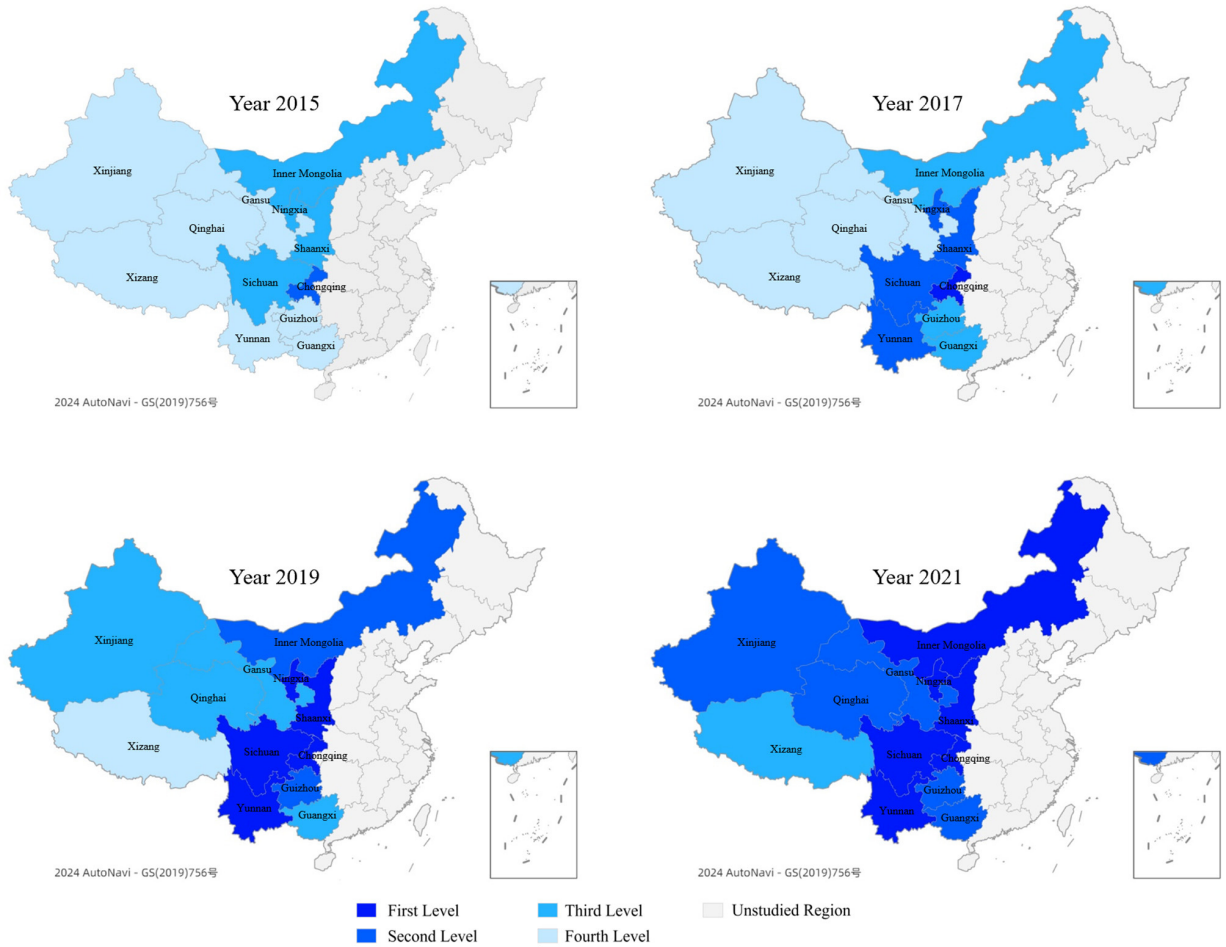
Spatial pattern level distribution of veterinary talent competitiveness. First level, high level, score ranges 3.717–5.811; Second level, relatively high level, score ranges 3.003–3.716; Third level, relatively low level, score ranges 2.225–3.002; and Fourth level, low level, score ranges 0.620–2.224.

According to the principle of quartile difference in statistics, the veterinary talent competitiveness in 12 regions from 2015 to 2021 is divided into four levels based on their scores: the first level (high level), the second level (relatively high level), the third level (relatively low level), and the fourth level (low level). Their respective score ranges are 3.717–5.811, 3.003–3.716, 2.225–3.002, 0.620–2.224. This study selected data from 2015, 2017, 2019, and 2021 for comparison, and use ArcGIS software to draw a spatial pattern level distribution of veterinary talent competitiveness (see Figure 2).

This study found that the overall pattern for veterinary talent competitiveness in Western China has undergone significant changes, with all regions maintaining an upward trend, extending from northeast to southwest. Chongqing's competitiveness has always been the strongest, while Xizang's has always been the weakest. Compared with 2015, the number of regions with the first and second levels of competitiveness in 2021 has significantly increased, from 0 and 1 to 6 and 5, respectively. Most regions have increased by 2 levels. The most significant change is in Yunnan, which has risen from the fourth level to the first level, with an increase of three levels. The competitiveness of Xizang has improved slowly, from the fourth level to the third level, with only one level of improvement.

#### 3.3.2 Spatial autocorrelation analysis of veterinary talent competitiveness

Based on Equations 8, 9, the global spatial autocorrelation analysis and significance test were conducted on the veterinary talent competitiveness using the ArcGIS software (see Table 4).

**Table 4:** Global spatial autocorrelation of veterinary talent competitiveness.

	**2015**	**2017**	**2019**	**2021**
I Value	0.2791	0.3276	0.3024	0.2286
Z-Score	2.0127	2.2912	2.0956	1.7021

From Table 4, the Global Moran's I value of veterinary talent competitiveness over the years are all >0, showing a significant positive spatial correlation feature in the veterinary talent competitiveness. Each region presents a clustering phenomenon in space. That is, there are one or several regions with higher competitiveness around regions with higher competitiveness, and regions with lower competitiveness will also have one or several regions with lower competitiveness adjacent to them. Using Z-Score to test the significance of the index, it was found that the Z-Score in 2015, 2017, and 2019 were greater than the critical value of 1.96, indicating a significant spatial autocorrelation and a clustering distribution of veterinary talent competitiveness. The Z-Score in 2021 is all < 1.96, indicating a random distribution of observed values. Although there were fluctuations, the I values remained above 0.2, maintaining a relatively stable spatial pattern overall.

**Figure 3:**
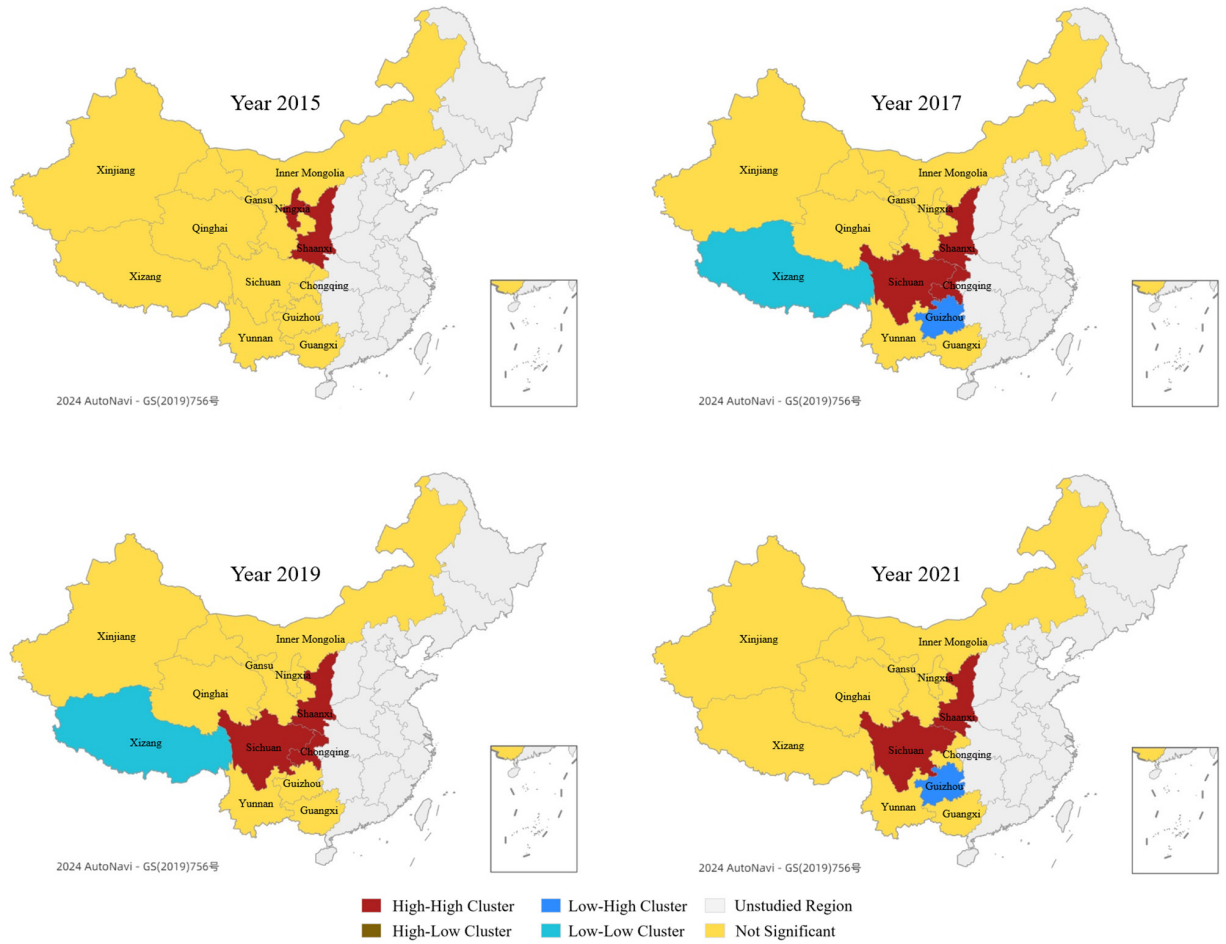
LISA analysis map of veterinary talent competitiveness.

Based on Equation 10, this study uses ArcGIS software to conduct LISA analysis on the veterinary talent competitiveness in 12 regions. The spatial agglomeration types were visualized to obtain the LISA analysis map of talent competitiveness (see Figure 3).

Figure 3 show that the regions with “H-H” clustering characteristics in veterinary talent competitiveness are concentrated in regions such as Chongqing, Sichuan, and Shaanxi. The regions with “L-L” clustering characteristics are only Xizang, and the regions with “L-H” clustering characteristics are only Guizhou, without any regions with “H-L” clustering characteristics.

There is a spatial agglomeration of veterinary talent competitiveness in Western China with increasingly evident regionalization and a relatively stable spatial pattern. The competitiveness remains on the rise, extending from northeast to southwest. The spatial differentiation of talent competitiveness has shifted from dispersion to aggregation, and the regionalization of aggregation has become increasingly evident. A clear positive spatial correlation characteristic indicates a stable overall spatial pattern. Regarding evolutionary trends, the local spatial correlation characteristics of veterinary talent competitiveness have not changed much, with severe spatial differentiation and no spatial linkage pattern yet formed, indicating that the regional veterinary talent competitiveness has certain path dependence or spatial locking characteristics.

## 4 Discussion

This study is based on veterinary education and the characteristics of veterinary talent. A veterinary talent competitiveness evaluation index was developed, incorporating the Entropy method and ArcGIS software. These methods were utilized to analyze the level and spatiotemporal evolution of veterinary talent competitiveness in Western China from 2015 to 2021. Based on the previous analysis, the main discussions of this study are as follows:

The weightings of dimensions and indicators for evaluating the veterinary talent competitiveness indicate that talent resources exert the greatest influence on this competitiveness. Specifically, the number of veterinary talents, particularly the proportion of those holding senior technical titles and master's degrees or above, exerts a notable impact. Talent resources constitute the most important part of veterinary talent competitiveness, which is consistent with the research results on agricultural technology talent competitiveness (84). Talent resources refer to the group of people with higher levels of quality in human resources (66), reflecting the quantity, quality, and structure of talent (63, 85), it is the most fundamental factor affecting talent competitiveness (86). A sufficient number of talent is the foundation of competitive advantage, ensuring initiative in industrial development (87), and the quality of talent is crucial for high-performance human resources (88, 89). Given the significant impact of high-quality talent on the competitiveness of veterinary professionals, it is important to focus on increasing the quantity and quality of veterinary professionals. Targeted education reform and talent development strategies can enhance the quantity and quality of senior talents in the veterinary industry (90, 91). Veterinary education should strengthen employment skills and practical experience, promote cooperation with employers, and improve the quality and retention rate of veterinary professionals (9, 92), contemporary practices and technologies should also be incorporated to align with the Industry 4.0 trend (93). By providing advanced training and continuing education in the field of veterinary medicine, professionals can be informed of the latest developments (90), enhance their professional knowledge, and promote innovation (92). Attractive salary and clear career paths also help attract and retain top talents in the veterinary field (94), achieving sustainable development of industry talent (95). In addition, universities should provide graduate education that focuses on cultivating future abilities, consistent with regional talent development strategies (96).

The veterinary talent competitiveness in western regions has been improving year by year, but there is still significant room for improvement. In recent years, China's economy and society have developed rapidly, and the strategies of western development and rural revitalization have been deeply implemented (97). The continuous improvement of various indicators in four dimensions has enabled the veterinary talent competitiveness to be continuously enhanced. Talent competitiveness is a complex, multidimensional, and relative concept (98), which comprehensively considers the level of human resource development (33). The government should develop talent development strategies that focus on attracting, developing, and retaining talent (86), accelerate the transformation of the population into talent that meets the requirements of industry enterprises (99), thereby enhance the talent competitiveness.

The uneven development of veterinary talent competitiveness in different regions is related to local education resource allocation, policy support, talent cultivation, and economic development level (100–102). Targeted strategies are needed to improve the competitiveness of regions with lower competitiveness (103). Implementing policies that focus on developing human capital potential can enhance the talent competitiveness of underdeveloped regions (104). Education is a long-term investment (70), and from an educational perspective, talent development requires high-quality education (105). In the regional innovation ecosystem, higher education institutions play a crucial role in developing, attracting, and retaining talent (106). To attract and retain talent, the government needs to invest more resources in higher education (107), increase support for education in relatively underdeveloped areas (108), and actively improve education infrastructure in underdeveloped regions (109). Universities can also rely on their majors to introduce veterinary industry enterprises to invest equipment and improve the practical training scenarios for veterinary students (110).

The spatial agglomeration trend of veterinary talent competitiveness in western regions continues to strengthen, maintaining a relatively stable spatial pattern, but a spatial linkage pattern has not yet been formed. This result is similar to the agglomeration of talent competitiveness in China (111). There is a spatial agglomeration effect in competitiveness (112), and regions with better economic development have a higher degree of agglomeration (113, 114). The spatial agglomeration effect of regions with stronger competitiveness should be leveraged to enhance their radiation and driving effect on regions with weaker competitiveness (115, 116). The trend of spatial clustering suggests that in the future, efforts can be made to establish regional centers of excellence in veterinary science, promoting technical exchange and collaborative innovation in professional fields (117). In addition, cooperation among different regions should be encouraged, as best practices and resource aggregation can help alleviate spatial disparities (118, 119), and enhance the overall competitiveness of veterinary talents.

## 5 Conclusion

Improving the quantity and quality of veterinary talent cultivation is an urgent issue to address in current veterinary education and is key to enhancing veterinary talent competitiveness. Introducing scientific analytical methods for studying veterinary talent competitiveness provides a new perspective for veterinary education. This study develops a veterinary talent competitiveness evaluation index based on the characteristics of veterinary talent. It proposes a visual method to analyze the level and spatiotemporal evolution of veterinary talent competitiveness using the Entropy method and ArcGIS tools, with Western China as a case study. The results show that this method not only evaluates the current state of veterinary talent competitiveness but also considers temporal and spatial evolution, achieving good evaluation effectiveness and high accuracy, thereby guiding the improvement of veterinary education and talent cultivation.

This study proposes a scientific method for evaluating the level and spatiotemporal evolution of talent competitiveness in the veterinary industry, providing new ideas and methods for quantitative research and visual display of the competitiveness, expanding the field of veterinary research, and promoting interdisciplinary research in veterinary science, economics, and geography. It has theoretical significance and methodological contributions. In addition, this study helps to accurately grasp the demand for veterinary talents, assist governments and educational institutions in adjusting veterinary education plans and strategies, ensure that education content and direction are synchronized with industry demand, cultivate talents that meet market demand, further optimize and improve education quality, and achieve a virtuous cycle between veterinary education and industry development, which has practical significance.

However, as various factors influence industrial talent competitiveness, we plan to incorporate more elements affecting veterinary talent competitiveness in the future to optimize the evaluation index continuously. Meanwhile, continuous monitoring of the spatial and temporal evolution of veterinary talent competitiveness is essential for understanding and responding to the constantly changing demands of the veterinary industry. In addition, we will introduce machine learning algorithms and more econometric models to evaluate and predict veterinary talent competitiveness, promoting the improvement of veterinary education and veterinary talent cultivation quality.

## Data Availability

The original contributions presented in the study are included in the article/supplementary material, further inquiries can be directed to the corresponding authors.
